# Dr. Webster W. Wynn

**Published:** 1908-04

**Authors:** 


Dr. Webster W. Wynn, of Dixon, Ill., died at his home February 28, 1908, of paralysis, aged 78 years. He graduated from the University of Buffalo in 1855, and was a post surgeon at Dixon during the civil war.

